# Fibrosis Distinguishes Critical Limb Ischemia Patients from Claudicants in a Transcriptomic and Histologic Analysis

**DOI:** 10.3390/jcm9123974

**Published:** 2020-12-08

**Authors:** Guangzhi Cong, Xiangdong Cui, Ricardo Ferrari, Iraklis I. Pipinos, George P. Casale, Ansuman Chattopadhyay, Ulka Sachdev

**Affiliations:** 1 Department of Surgery, University of Pittsburgh Medical Centre, Pittsburgh, PA 15217, USA; congg@upmc.edu (G.C.); cuix@upmc.edu (X.C.); ferrarirj2@upmc.edu (R.F.); 2 Department of Surgery, University of Nebraska at Medical Center, Omaha, NE 68198, USA; 3 Department of Surgery and VA Research Service, VA Nebraska-Western Iowa Health Care System, Omaha, NE 68198, USA; ipipinos@unmc.edu (I.I.P.); gpcasale@unmc.edu (G.P.C.); 4 Molecular Biology Information Service, Health Sciences Library System University of Pittsburgh, Pittsburgh, PA 15261, USA; ansuman@pitt.edu

**Keywords:** peripheral artery disease (PAD), transcriptomics, fibrosis pathway, claudication, critical limb ischemia (CLI)

## Abstract

Most patients with critical limb ischemia (CLI) from peripheral arterial disease (PAD) do not have antecedent intermittent claudication (IC). We hypothesized that transcriptomic analysis would identify CLI-specific pathways, particularly in regards to fibrosis. Derivation cohort data from muscle biopsies in PAD and non-PAD (controls) was obtained from the Gene Expression Omnibus (GSE120642). Transcriptomic analysis indicated CLI patients (*N* = 16) had a unique gene expression profile, when compared with non-PAD controls (*N* = 15) and IC (*N* = 20). Ninety-eight genes differed between controls and IC, 2489 genes differed between CLI and controls, and 2783 genes differed between CLI and IC patients. Pathway enrichment analysis showed that pathways associated with TGFβ, collagen deposition, and VEGF signaling were enriched in CLI but not IC. Receiver operating curve (ROC) analysis of nine fibrosis core gene expression revealed the areas under the ROC (AUC) were all >0.75 for CLI. Furthermore, the fibrosis area (AUC = 0.81) and % fibrosis (AUC = 0.87) in validation cohort validated the fibrosis discrimination CLI from IC and control (all *n* = 12). In conclusion, transcriptomic analysis identified fibrosis pathways, including those involving TGFβ, as a novel gene expression feature for CLI but not IC. Fibrosis is an important characteristic of CLI, which we confirmed histologically, and may be a target for novel therapies in PAD.

## 1. Introduction

Peripheral artery disease (PAD) is a manifestation of systemic atherosclerosis with associated adverse cardiovascular mortality and risk of amputation [1]. According to the latest version of Heart Disease and Stroke Statistics reported by the American Heart Association, 6.5 million Americans aged ≥40 years (5.5%) are estimated to be afflicted with PAD [2]. Among them, 2 million individuals are currently suffering from critical limb ischemia (CLI) [3,4], which represents the most severe form of PAD. CLI is traditionally viewed as a progressive continuum from intermittent claudication (IC). However, most IC patients do not progress to CLI [5,6], which suggests that CLI may represent a unique feature of PAD with distinct etiology.

CLI is diagnosed based on clinical presentation of either tissue loss or rest pain, coupled with hemodynamic measurements of ankle bronchial index (ABI) or toe pressures. The anatomic location of vascular disease is confirmed with traditional or computed tomographic, with occasional use of magnetic resonance imaging (MRI) [7]. However, as stated by ‘’Critical Limb Ischemia: An Expert Statement’’, approximately 30% of patients with CLI have a near-normal or normal ABI (>0.90) [7], possibly due to vessel calcification [8,9]. This suggests there is significant heterogeneity of pathogenesis among PAD patients. The use of transcriptomic data provides promising workflows for personalized diagnosis and treatment of cardiovascular diseases in several landmark studies [10,11,12,13]. Because transcriptome-wide profiling enables us to detect novel, un-annotated genes, sequence-level alterations, and alternative splicing events, it offers greater information about tissue than microarrays. We, therefore, hypothesized that transcriptomic analysis will identify CLI-specific pathways, particularly in regards to fibrosis, that will distinguish critical limb ischemia patients from those with intermittent claudication.

To address this hypothesis, we performed an analysis of a publicly available and recently published RNA-sequencing database of PAD cohorts. Following this, we evaluated the diagnostic performance of fibrosis pathway gene signatures for distinguishing CLI from IC and validated the findings using fibrosis staining of skeletal muscle biopsies of PAD patients. 

## 2. Experimental Section

### 2.1. Study Design

**Figure 1:**
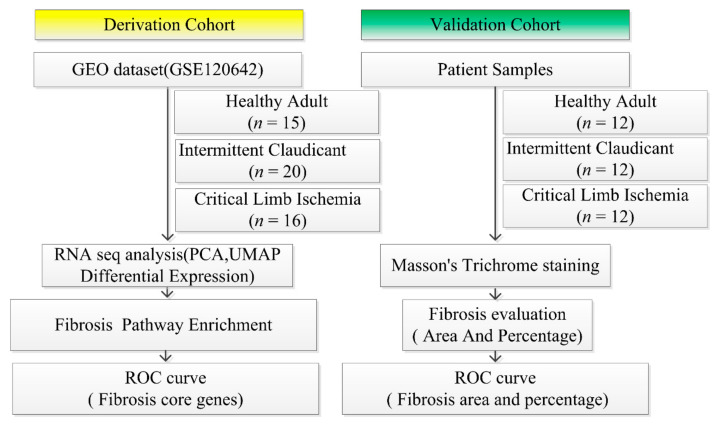
Flow Diagram of Study design and data processing. RNA sequence data of 51 skeleton muscle biopsies in peripheral arterial disease (PAD) (16 of critical limb ischemia (CLI) and 20 of intermittent claudication (IC)) and non-PAD controls (*N* = 15) were retrieved from the Gene Expression Omnibus (GEO) database (GSE120642). After principal component analysis, Uniform Manifold Approximation and Projection (UMAP), differential expression analysis, hierarchical cluster analysis, and pathway enrichment analysis were completed using Ingenuity Pathway Analysis (http://www.ingenuity.com). The ability of fibrosis modular gene signatures to discriminate CLI from IC was assessed by ANOVA and receiver operating curve (ROC). For validation, we evaluated fibrosis in 36 muscle biopsies from non-PAD controls, IC, and CLI patients using Masson’s trichrome staining. After the measurement of fibrosis area and percentage, we conducted ROC analysis. PCA, principal component analysis.

An observational, cross-sectional study was conducted to explore whether fibrosis identified by transcriptomic and histology assay can distinguish CLI patients from IC. We reasoned that RNA-sequencing could provide insight into a pathogenesis pathway and could potentially be a complementary diagnostic tool. To interpret transcriptional differences seen in patients with and without PAD, transcriptome profiles of PAD patients and non-PAD controls were obtained from the Gene Expression Omnibus (GEO; GSE120642) resulting from studies by Ryan et al. [14]. First, transcriptomic profiles that distinguished CLI patients from those with claudication and controls were identified. We then validated our findings using histologic techniques to confirm fibrosis (Figure 1).

### 2.2. Retrieval of Derivation Cohort Datasets of Patients from the GEO

A derivation cohort of PAD patients and control subjects without PAD with RNA-sequencing-based profiles were acquired from the public functional genomics data repository GEO and analyzed [14]. In this published cohort, gastrocnemius muscle was obtained from PAD patients and non-PAD controls for whole transcriptome sequencing. The diagnosis of IC and CLI was based on clinical characteristics and ankle-brachial index [14]. The diagnostic criteria and participant’s demographic and medical information were explicitly illustrated in the paper [14].

### 2.3. Bioinformatics Analysis

Paired FASTQ files storing sequencing data from gastrocnemius muscle was obtained [14]. RNA-seq data analysis was done using CLC Genomics Workbench v20.0 (Qiagen Digital Insights, Aarhus, Denmark)—a software suite that provides a variety of tools for next-generation sequence analysis. The RNA-seq analysis workflow, as described in the CLC Genomics manual, was followed [15]. Briefly, single-end RNA-seq reads, obtained in FASTQ format, were checked for sequence quality using the tool “QC for Sequencing Reads” available under the toolbox item “Prepare Sequencing Data.” Reads with Phred quality score > 20 were then aligned to the human reference genome GRCh38 assembly sequence using the default parameters provided by the “RNA-seq Analysis” tool available under the “RNA-Seq Analysis” toolbox item. Genes and transcripts annotations provided by Ensembl (release 92) were used. Around 94% of reads from each sample were mapped into the reference genome [15,16].

To explore transcriptomics features among subtype of PAD, we conducted cluster analysis and dimensional reduction analysis by principal component analysis (PCA), uniform manifold approximation and projection (UMAP), and hierarchical cluster analysis. Empirical analysis of differentially expressed reads was conducted to identify differentially expressed genes (DEGs) with default parameters. DEGs were filtered according to pairwise comparisons for biological and statistical significance (fold change (FC) > 2, false discovery rate (FDR) < 0.05) [16].

### 2.4. Pathway Enrichment Analysis

To explore the functional enrichment of biological pathways and upstream molecules that are enriched in DEGs, pathway enrichment analysis and visualization was performed using IPA (QIAGEN Inc., Redwood City, CA, USA). Briefly, IPA regulation z-score algorithm will be used to determine the activation state of canonical pathways and upstream regulators using the general molecular network implemented in the ingenuity pathway analysis (IPA) knowledge base. To enhance the stringency of our analysis, only z-score >2 or <−2 (two folds) is considered significant as positive or negative [17].

### 2.5. Fibrosis Core Gene Expression Analysis

Transforming Growth Factor-β, collagen deposition, and vascular endothelial growth factor (VEGF) signaling pathway are the main contributors to the formation of fibrosis [18]. Therefore, the fibrosis core gene expression in skeletal muscle transcriptomics, including TGFβ1, TGFβ2, TGFβ3, COL1A1, COL1A2, VEGFA, VEGFB, VEGFC, and VEGFD were counted specifically as total counts and Transcripts Per Kilobase Million (TPM). The calculation and visualization were run by CLC Genomics Workbench 20.0 (Qiagen Digital Insight, Aarhaus, Denmark) and Partek^®^ Flow^®^ software, version 7.0 Copyright©; 2020 Partek Inc., St. Louis, MO, USA.

### 2.6. Validation Cohort Data

Validation cohort sets of gastrocnemius muscle biopsies slides were obtained from the University of Nebraska Medical Center (UNMC). The diagnosis of IC and CLI was also based on clinical characteristics and ankle-brachial index. Biopsy techniques and tissue processing were performed as described previously using a 6 mm Bergstrom needle under anesthesia [19]. Specimens were paraffin-fixed and sequentially sectioned. Masson’s trichrome staining was performed to identify areas of fibrosis.

### 2.7. Ethical Compliance

The study complied with all relevant ethical regulations consistent with the policies of the Omaha VA Medical Center, the University of Nebraska Medical Center, and the University of Pittsburgh Medical Center. The study guiding the procurement of muscle tissue was approved by the IRB of the Omaha VA Medical Center in accordance with the Declaration of Helsinki (ID# 00086, Protocol #01), and all patients provided written informed consent.

### 2.8. Fibrosis Evaluation in Validation Cohort

The fibrosis levels of gastrocnemius muscle biopsies were determined by Masson’s trichrome staining [20]. Photographs were taken with Olympus Provus-1 fluorescence microscope using a 20X objective. To quantify the extent of fibrosis, stained sections were analyzed using Image J analysis program [21] after color separation of the figures [22]. Percent and area of fibrosis were determined based on the number of pixels per image that were positive for fibrosis-specific staining (blue) in each section, represented as a percentage of the total area. Images were analyzed in a blinded fashion.

### 2.9. ROC Analysis Both in Derivation and Validation Cohort

To determine the specificity and sensitivity of the fibrosis pathway, receiver operating curve (ROC) analysis of the expression of nine core fibrosis genes in a derivation cohort was performed. Then, ROC analysis of percentage and area of fibrosis was performed in the validation. Specificity was designated on the x-axis, and sensitivity was designated on the y-axis. An area under the curve (AUC) = 1.0 suggests an ideal test, while an AUC < 0.5 indicates a non-diagnostic test.

### 2.10. Statistics for Clinical Data

Categorical variables were compared between non-PAD controls, IC, and CLI group using Χ^2^ test. Continuous variables were compared between HA, IC, and CLI group using single one-way analysis of variance (ANOVA) followed by Bonferroni t-test for data involving more than two groups. All *p* values reported are considered to be descriptive, and no adjustments for multiple comparisons were made. Statistical software package R-3.4.3 [23], The R Foundation) and Empower-Stats (X&Y Solutions, Inc., Boston, MA, USA) were used for the demographic analyses.

## 3. Results

### 3.1. Subjects’ Age and ABI Derivation and Validation Cohort

Table 1 demonstrates clinical characteristics of the PAD and control subjects from the derivation and validation cohorts.

The results of derivation cohort cited to published paper in JCI insight [14].

### 3.2. CLI Patients Have a Unique Gene Expression Profile Compared to Claudicants and Non-PAD Controls Subjects

**Figure 2:**
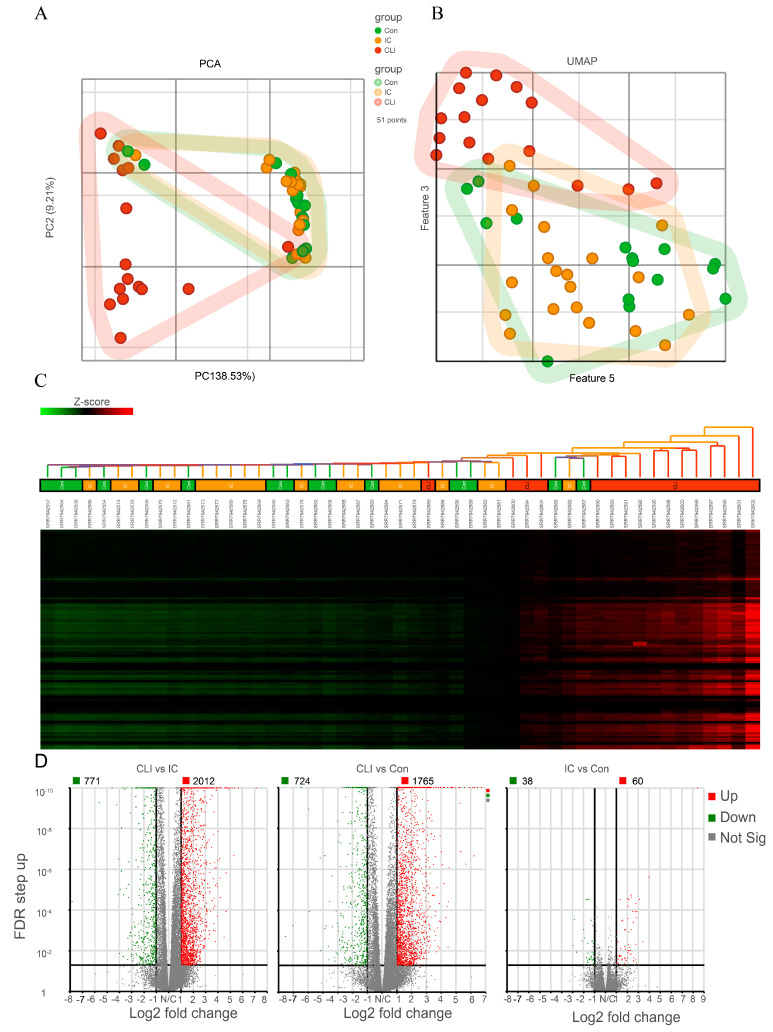
Unique gene expression profile presenting in CLI patients. (**A**) Principal component analysis (PCA) of the samples. Each dot represents one subject. (**B**) (UMAP) of the samples. Each dot represents one subject. (**C**) Heatmap of all differentially expressed genes among groups. Samples are on columns, genes are on columns, and the heat map is based on standardized gene expression values. (**D**) Volcano plot representing differential gene expression among groups. Each dot on the plot is a single gene. Horizontal axis: fold change; vertical axis: false discovery rate (FDR) *p*-value (in log10 scale) by Wald test. Color coding is based on the fold change. Thick vertical lines highlight fold changes of −2 and +2, while a thick horizontal line represents a FDR *p*-value of 0.05. *N* = 15 for non-PAD controls, *N* = 20 for IC, *N* = 16 for CLI. PC1, principal component; CLI, critical limb ischemia; IC, intermittent claudication.

To explore the transcriptomic features in these two different clinical manifestations of PAD, we conducted a dimensionality reduction by PCA, UMAP, and hierarchical cluster analysis. As shown in Figure 2A,B, PCA and UMAP revealed that CLI samples clustered in a distinct pattern from both control and IC patients. Meanwhile, control and IC patients’ transcriptomic profiles overlapped significantly. By hierarchical cluster analysis and differential expression analysis (DEA), we observed that 2783 genes out of 29,191 total genes (2012 up-regulated, and 771 down-regulated) were differentially expressed in CLI when compared to IC (Figure 2C,D), and 2489 genes out of 29,191 total genes (1765 up-regulated, and 724 down-regulated) were differentially expressed in CLI when compared to control (Figure 2D). However, there are only 98 genes out of 29,191 total genes (60 up-regulated, and 38 down-regulated) that were differentially expressed in IC when compared to control (Figure 2C,D).

### 3.3. Differentially Expressed Genes (DEG) and Canonical Pathway Analysis Distinguishes CLI Patients

**Figure 3:**
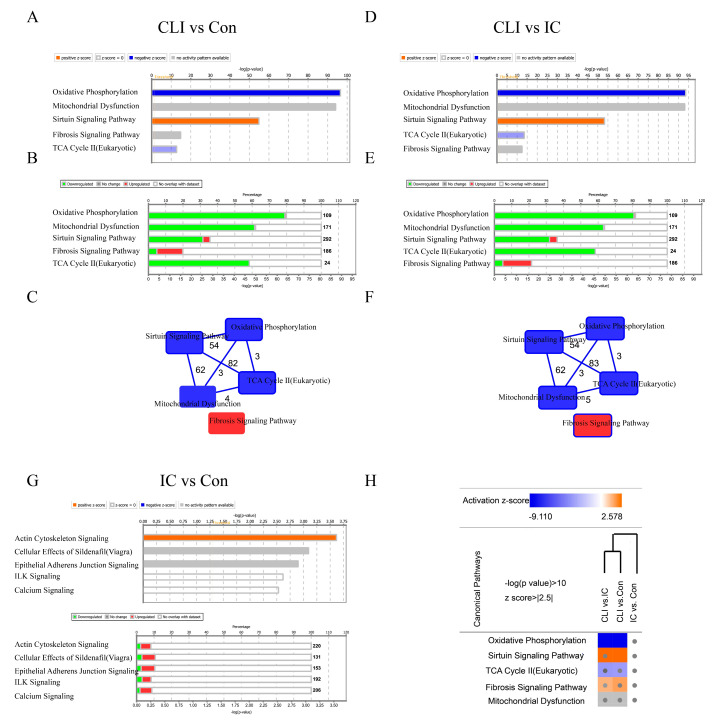
IPA of genes differentially expressed between CLI and IC and Con. Differentially expressed genes calculated using DeSeq2 (FC > 2, FDR < 0.05) were merged and submitted for IPA analysis. (**A**,**D**) A selection of highly significant functions and their activation z-scores are shown in the bar graphs (orange for positive Z score, blue for negative Z score, and gray for no activation pattern available). The x-axis corresponds to the –log of the *p*-value (Fisher’s exact test); (**B**,**E**) a selection of highly significant functions and their activation percentage are shown in the stack bar graphs (red for activation, green for inhibition. Number for each bar for the total molecular in this pathway.) (**C**,**F**) Overlaying pathways. (**C**) CLI vs. Con; (**F**) CLI vs. IC; (**A**–**C**) for CLI vs. Con; (**D**–**F**) for CLI vs. IC. (**G**) Bar and stacked bar graph of first five function and their activation when IC vs. Con. (**H**) Canonical pathway heatmap of three comparisons. 3.4 Fibrosis pathways are enriched in CLI patients compared to IC and controls. TCA, Tricarboxylic Acid Cycle; FC, Fold Change; ILK, Integrin Linked Kinase; IPA, Ingenuity Pathway Analysis.

A nonbiased analysis of the canonical pathways enriched by the differentially expressed genes revealed that oxidative phosphorylation, mitochondrial dysfunction, sirtuin signaling pathway, TCA cycle II, and fibrosis signaling pathways were distinct in CLI when compared with IC and controls (Figure 3A,B,D,E). Among these five canonical pathways, the fibrosis pathway and sirtuin signaling were activated, while the other pathways were inhibited. By overlaying these pathways, we found that oxidative phosphorylation, mitochondrial dysfunction, sirtuin signaling pathway, and TCA cycle II were connected to each other and shared common genes. On the contrary, the fibrosis pathway did not share common genes with the other pathways (Figure 3C,F). Therefore, we selected fibrosis to determine whether it was a candidate pathway differentiating CLI from IC and controls. Furthermore, there is only activation of actin cytoskeleton signaling with a small proportion of genes involved as showed in Figure 3G. Canonical pathway heatmap of three comparisons confirms that fibrosis pathway was activated in CLI but not IC when compared with non-PAD patients (Figure 3H).

**Figure 4:**
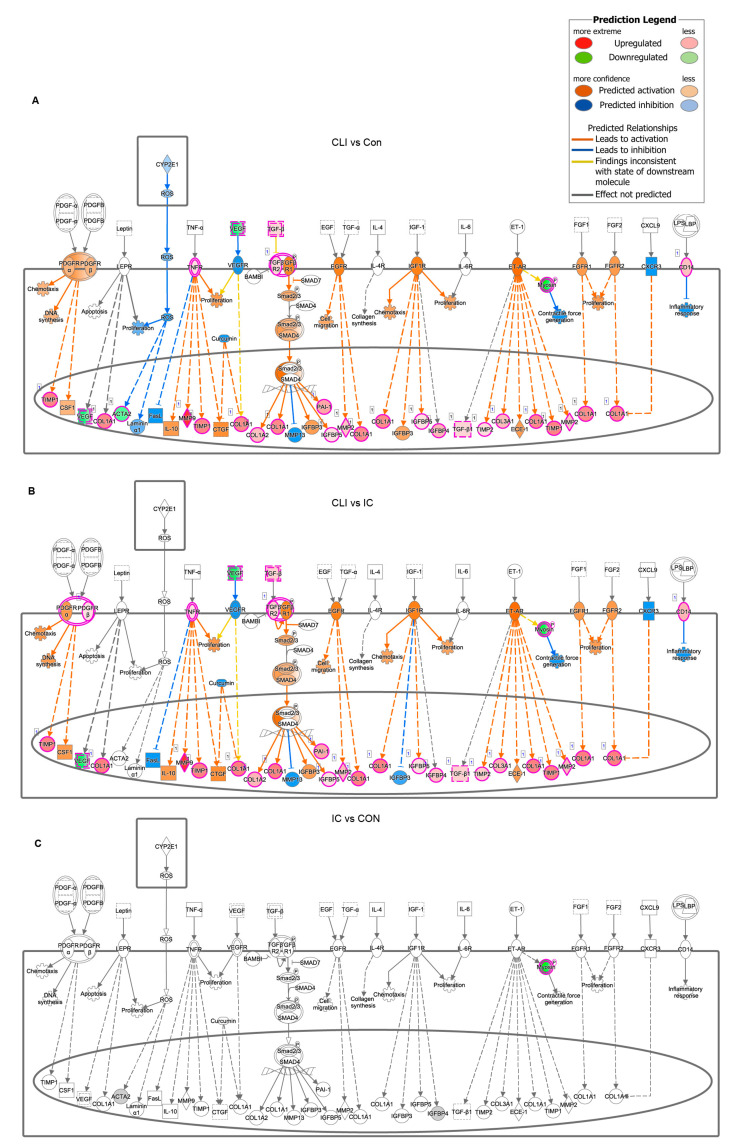
Network overview of modular expression patterns of fibrosis pathway. (**A**) CLI vs. Con; (**B**) CLI vs. IC; (**C**) IC vs Con. Ingenuity Pathway Analysis of the differentially regulated genes when CLI vs. control and CLI vs IC. The network is displayed graphically as nodes (genes/gene products) and edges (biological relationship between nodes). The node color intensity indicates the fold change expression of genes, with red representing activation, green representing down-regulation of genes, orange representing prediction activation, and blue representing prediction inhibition; the lines indicate the type of interaction.

To illustrate the potential differences in the canonical fibrosis pathway in CLI patients, we plotted the core molecular networks that may constitute the fibrosis pathway by IPA software, which compares Genbank Accession number/expression information with a proprietary protein-interaction database to establish the probability of a given signaling. When comparing CLI samples to controls, gene clusters that participate in TGFβ signaling, collagen deposition, VEGF pathways, and DNA synthesis were either widely activated or predicted to be activated (Figure 4A). Similarly, TGFβ signaling, collagen deposition, and VEGF pathways were activated in CLI compared to IC (Figure 4B). In contrast, these pathways did not differ when IC was compared to control patients (Figure 4C).

### 3.4. Fibrosis Pathway Core Genes Expression in Transcriptomics and ROC in the Derivation Cohort

**Figure 5:**
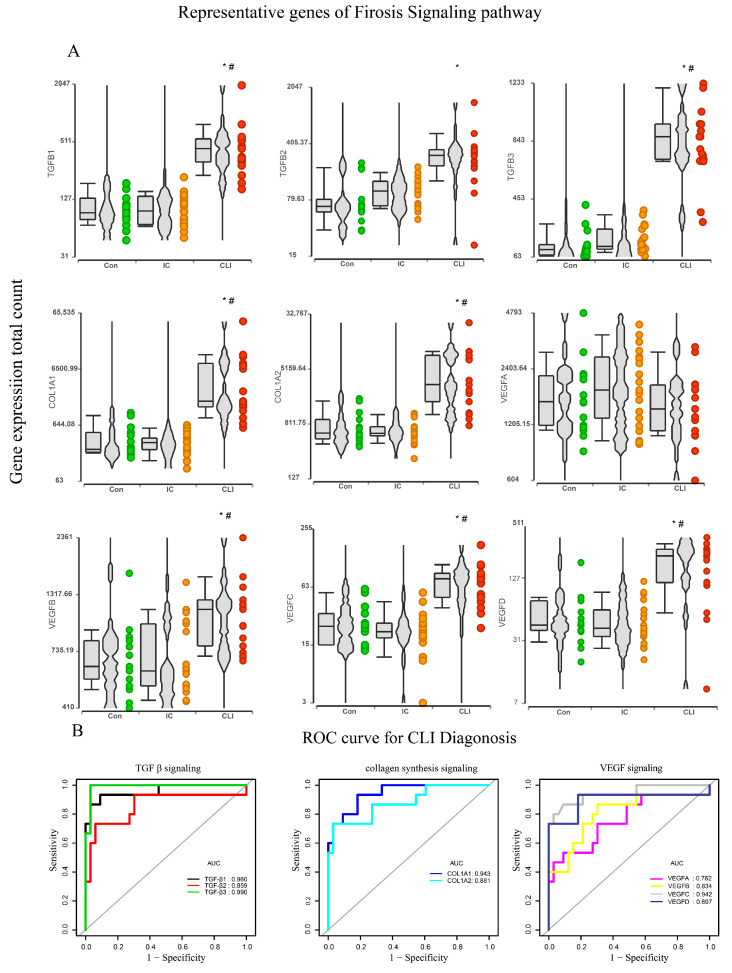
Fibrosis pathway core genes distinguish CLI from IC and control. (**A**) Representative gene expression of fibrosis pathway by transcriptomic by box and whiskers, violins, and points plot; each dot is a sample. The plot title is the gene symbol of the selected gene. Expression levels are log (total counts) on the vertical axis. * *p* < 0.05, compared with non-PAD controls by wald test, ^#^ *p* < 0.05, compared with IC by wald test. (**B**) Receiver operating curve (ROC) (spell out ROC) for the diagnosis of CLI of TGF β signaling, collagen deposition signaling, and vascular endothelial growth factor (VEGF) signaling. All area under curve (AUC) > 0.75. *N* = 15 for non-PAD control, *N* = 20 for IC, and *N* = 16 for CLI.

TGFβ signaling, collagen synthesis, and deposition and VEGF signaling are key components of healing and fibrosis and likely coordinate with each other in the formation of skeletal muscle fibrosis [24]. The gene expression of core genes of TGFβ signaling (TGFβ1, TGFβ2, and TGFβ3), collagen synthesis and deposition (COL1A1 and COL1A2), and VEGF signaling (VEGF A, VEGF B, VEGF C, and VEGF D) were therefore calculated and expressed as total counts and TPM by CLC Genomics Workbench 20.0. Most of the core genes were activated when comparing CLI to IC and controls (Figure 5A). We then evaluated the diagnostic performance of these nine core genes to discriminate CLI from IC and controls using AUC. As shown in Figure 5B, the TGFβ signaling (TGFβ1, TGFβ2, and TGFβ3), collagen synthesis (COL1A1 and COL1A2) and VEGF signaling (VEGFA, VEGFB, EGFC, and VEGFD) yielded good discriminatory power (all AUC > 0.75).

### 3.5. Fibrosis Quantification and ROC in the Validation Cohort

**Figure 6:**
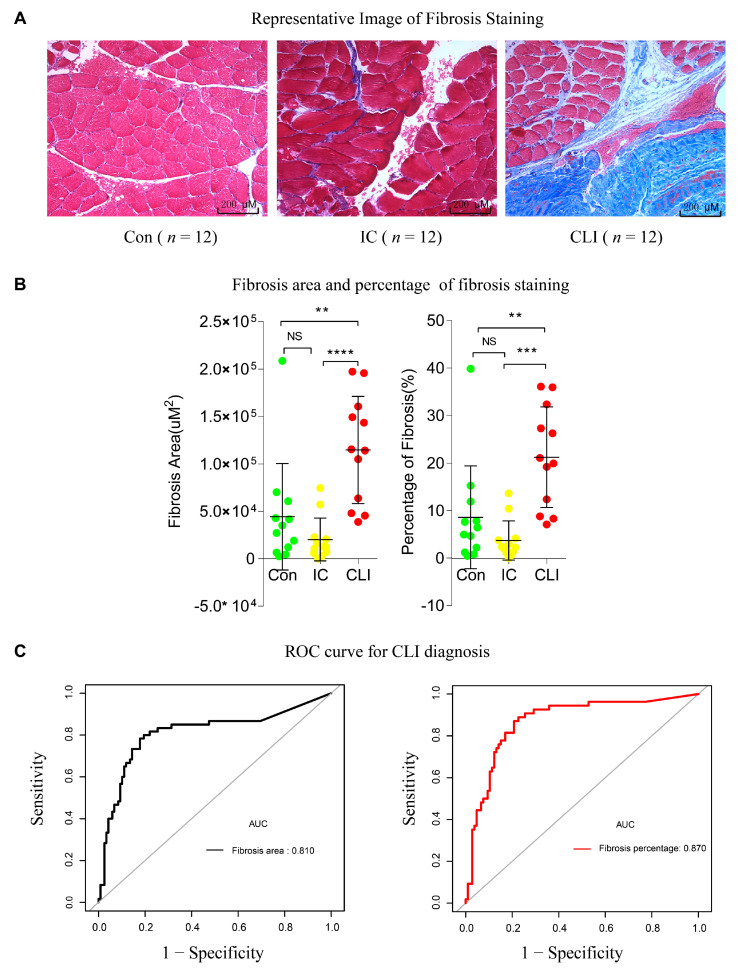
Validation of fibrosis pathway to distinguish CLI from IC and control. Masson trichrome staining of gastrocnemius muscle biopsies. (**A**) Representative fibrosis staining image (×20 magnification). (**B**) Assessment of fibrosis by area and percentage. Each dot is a sample. Comparison between HA, IC, and CLI group using single one-way analysis of variance (ANOVA) followed by Bonferroni t-test; ** *p* < 0.01, *** *p* < 0.001, **** *p* < 0.0001. (**C**) ROC for the diagnosis of CLI. Fibrosis area (AUC area = 0.81) and fibrosis percentage (AUC area = 0.87). *N* = 12 for non-PAD control, IC, and CLI. NS, not significant.

To validate whether fibrosis was a prominent feature of CLI, we used Masson’s trichrome staining to detect fibrosis within muscle samples from CLI, IC, and control patients without PAD. Then, we accessed the area and percentage of fibrosis in patient’s biopsies slides and explored their discriminatory power for CLI. As depicted in Figure 6A,B, the fibrosis area and percentage were significantly increased in CLI compared to IC and control (all *p* < 0.05). Furthermore, the AUC of the fibrosis area and percentage were over 75%, which indicated that the discriminatory power of fibrosis is validated histologically (Figure 6C).

## 4. Discussion

**Figure 7:**
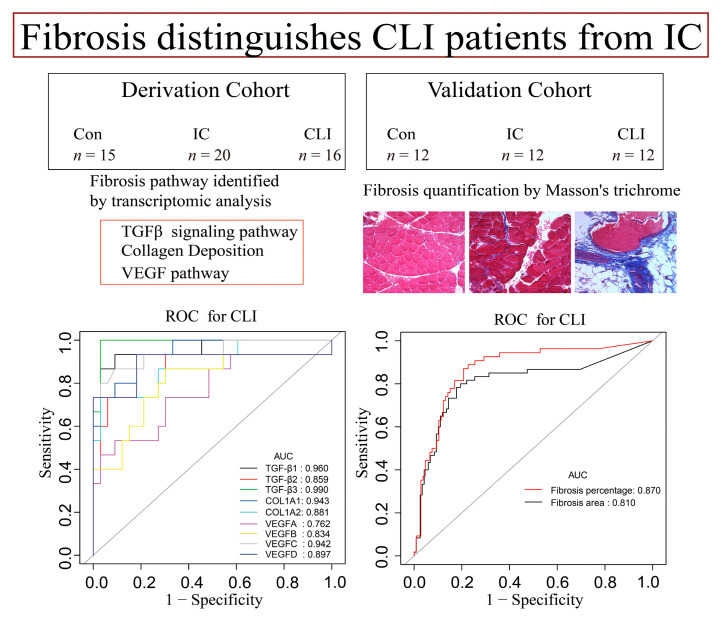
Derivation cohort included 15 non-PAD controls, 20 IC, and 16 CLI patients. Validation cohort included 12 non-PAD controls, 12 IC, and 12 CLI patients. Both fibrosis pathway identified by transcriptomics and fibrosis quantification by Masson trichrome of skeletal muscle demonstrated an area under the curve (AUC) over 0.75. This indicates that fibrosis distinguishes CLI patients from IC in a transcriptomic and histologic analysis.

CLI uncommonly develops as a progression of IC [5,6]. Instead, patients tend to present either with CLI or IC, suggesting that CLI has distinct pathogenesis from IC despite a similar ischemic etiology [24,25]. We used a transcriptomics approach to determine some of the identifying features of CLI and compared them to IC and normally perfused muscle. Previous PAD transcriptomic studies have not specifically defined gene expression levels and pathway analysis in relation to IC or CLI states. Sample sizes were also limited [26,27,28]. In this cross-sectional study, we found that (1) CLI patients present with a unique transcriptomic profile that is different from IC patients and non-PAD control; (2) fibrosis pathways that highlight TGFβ collagen deposition and VEGF distinguish CLI from IC and non-PAD control using transcriptomic data and IPA; and (3) increased fibrosis could be identified in CLI patients, validating the data we identified from the transcriptomic analysis (Figure 7). Our results provide a systems-level, data-driven determination of a complex network of molecular responses underpinning PAD, which was confirmed histologically. The data also provide important granularity for multiple distinct biological pathways, the relationships among these pathways, and their potential application in terms of the precision diagnosis and treatment of PAD.

Our findings are consistent with several proposed mechanisms of PAD progression, and confirm their importance in a relevant human study [29,30,31]. For example, TGFβ produced by vascular smooth muscle cells predicts fibrosis in the gastrocnemius of patients with PAD [30]. However, our study also determined other areas of difference between the groups that may be relevant. For example, our results suggest that there is inhibition of mitochondrial function in CLI. In contrast to claudicating and non-PAD control subjects, the pathways governing energy metabolism, including oxidative phosphorylation, mitochondrial dysfunction, sirtuin signaling, and TCA cycle II, were significantly suppressed. This is consistent with results in the study by Ryan et al. showing that skeletal muscle from CLI patients harbor a unique muscle mitochondriopathy when compared with IC patients [14]. Indeed, the RNA-sequencing data from the referenced study was made publicly available and was analyzed for this report. Muscle mitochondrial dysfunction has been demonstrated to be a key event in PAD [32]. Decreased oxidative capacity due to mitochondrial respiratory chain impairment is associated with an increased release of reactive oxygen species, leading to a reduction of calcium retention, and apoptosis [32]. However, detailing the intricacy of mitochondrial dysfunction is time-consuming and expensive, which may limit its utility in precision diagnosis and treatment [33,34].

Most notably, we identified fibrosis signaling as a critical event in CLI both by transcriptomic and histologic analysis. Fibrosis is the abnormal deposition of extracellular matrix, which is largely driven by activation of TGFβ signaling, collagen deposition, and VEGF signaling [18]. As shown in the fibrosis pathway enrichment analysis, these principal fibrosis component pathways were broadly activated. Ha et al. [30] reported that TGFβ signal was significantly activated in CLI patients and was generated in micro-vessels in response to tissue hypoxia. Our imaging was consistent with this study, and fibrosis in our images was often seen around vessels. Our observation may indicate a mechanism central to the association between ischemia and PAD progression [35] that relates to TGFβ activity; collagen deposition; and, ultimately, limb dysfunction.

Our results support the promising application of fibrosis signaling to distinguish CLI from IC. ABI is often the first-line noninvasive measure of limb ischemia in the clinic, but may be inaccurate if the blood vessels are very calcified [36]. Thus, there is a notable heterogeneity of ABI in patients with CLI [37]. Our results suggest that fibrosis may help distinguish the severity of PAD in a clinical setting, or can help guide future studies to mitigate disease. Fibrosis can be detected noninvasively using multiple imaging techniques including MRI [38]. We present that transcriptomic data may be able to identify activation of fibrotic pathways in patients with PAD. Transcriptomics has shown some promise in differentiating myopathies and muscular dystrophies in other clinical settings as described by Cummings [39]. Furthermore, transcriptomics of cardiac biopsies disclosed differences in patients with or without heart failure with preserved ejection fraction [40]. These studies consistently portrayed the potential of transcriptomic analysis to identify severity and pathogenesis in a variety of disease states, although its use in PAD is not optimized at this time.

### Strengths and Limitations

A major strength of our study is that all the data were derived from skeletal muscle biopsies of PAD and non-PAD patients instead of animal models, which facilitates translatability. By using transcriptome network analysis, we were able to investigate our aims in an unbiased manner, leading to the discovery of new findings while also confirming and expanding upon anticipated results. By using a bioinformatics network analysis validated histologically, we were able to distill a large dataset into a coherent, statistically rigorous, and biologically meaningful finding. However, our study also has important limitations. First, the study had a cross-sectional design, and specific interventions could not be studied. In the future, the application of transcriptomics can be performed before and after treatments including revascularization. Secondly, the study population we utilized is considered small from a clinical perspective with limited demographic and commodities information, which limits its generalization. Future clinical trials with a bigger sample size are needed to determine the chronology of budget constraints that usually exist and can significantly affect the trade-off decision between the sample size and sequencing depth [41]. Thus, reliance on GEO datasets, which are publicly available, allow for re-analysis of existing data to define potentially new avenues of further investigation. The biopsies that underwent staining for fibrosis were not obtained from the patients in whom the genetic analysis was performed, but rather were performed on a “validation” cohort of sorts. While specific staining for TGFb and other proteins highlighted by genetic analysis would have been ideal, our ability to do so was limited by the COVID-19 pandemic.

## 5. Conclusions

In summary, our results support that fibrosis identified by transcriptomic and histologic analysis enables us to distinguish CLI from IC. Fibrosis is an important consequence of CLI, which we confirmed histologically, and may be a target for novel therapies in PAD. This study demonstrates the power of RNA-sequencing to determine features that stratify risk and suggests potential therapy. This study further supports a transcriptomic approach to diseases along a spectrum such as PAD.

## Figures and Tables

**Table 1:** Patient characteristics.

	Non-PAD Controls	Intermittent Claudicant	Critical Limb Ischemia	*p* Value
**Derivation cohort**	*n* = 32	*n* = 27	*n* = 19	
Age (Mean ± SD)	61(7.3)	61(7.5)	64(10)	0.18
ABI (Mean ± SD)		0.65(0.21)	0.35(0.30)	0.0007
**Validation cohort**	*n* = 12	*n* = 12	*n* = 12	
Age (Mean ± SD)	66.75(4.93)	68.55(8.68)	63.58(5.05)	0.01
ABI (Mean ± SD)	1.10(0.10)	0.62(0.13)	0.31(0.24)	0.0001

PAD, peripheral artery disease; SD, standard deviation.

## Data Availability

All metadata from the study cohort have been deposited to Dryad Dataset (Cong, Guangzhi et al. (2020). Fibrosis distinguishes critical limb ischemia patients from claudicants in a transcriptomic and histologic analysis, Dryad, Dataset, [42]).
